# ‘The money is important but all women anyway go to hospital for childbirth nowadays’ - a qualitative exploration of why women participate in a conditional cash transfer program to promote institutional deliveries in Madhya Pradesh, India

**DOI:** 10.1186/s12884-016-0834-y

**Published:** 2016-03-04

**Authors:** Kristi Sidney, Rachel Tolhurst, Kate Jehan, Vishal Diwan, Ayesha De Costa

**Affiliations:** Karolinska Institutet, Public Health Sciences, Tomtebodavägen 18, Stockholm, 171 77 Sweden; Liverpool School of Tropical Medicine, Liverpool, L3 5QA Merseyside UK; R. D. Gardi Medical College, Agar Road, Surasa, Ujjain, 456006 Madhya Pradesh India

**Keywords:** Janani Suraksha Yojana, India, Maternal health, Conditional cash transfer

## Abstract

**Background:**

In 2005–06, only 39 % of Indian women delivered in a health facility. Given that deliveries at home increase the risk of maternal mortality, it was in this context in 2005, that the Indian Government implemented the Janani Suraksha Yojana program that incentivizes poor women to give birth in a health facility by providing them with a cash transfer upon discharge. JSY helped raise institutional delivery to 74 % in the eight years since its implementation. Despite the success of the JSY in raising institutional delivery proportions, the large number of beneficiaries (105 million), and the cost of the program, there have been few qualitative studies exploring why women participate (or not) in the program. The objective of this paper was to explore this.

**Methods:**

In March 2013, we conducted 24 individual in-depth interviews with women who delivered within the previous 12 months in two districts of Madhya Pradesh, India. Qualitative framework analysis was used to analyze the data.

**Results:**

Our findings suggest that women’s increased participation in the program reflect a shift in the social norm. Drivers of the shift include social pressure from the Accredited Social Health Activist (ASHA) to deliver in a health facility, and a growing individual perception of the importance for ‘safe’ and ‘easy’ delivery which was most likely an expression of the new social norm. While the incentive was an important influence on many women’s choices, others did not perceive it as an important consideration in their decision to deliver in a health facility. Many women reported procedural difficulties to receive the benefit. Retaining the cash incentive was also an issue due to out-of-pocket expenditures incurred at the facility. Non-participation was often unintentional and caused by personal circumstances, poor geographic access or driven by a perception of poor quality of care provided in program facilities.

**Conclusions:**

In summary, while the cash incentive was important for some women in facilitating an institutional birth, the shift in social norm (possibly in part facilitated by the program) and therefore their own perceptions has played a major role in them giving birth in facilities.

## Background

Each year 286,000 women die from childbirth globally [1]. An additional ten to twenty million women suffer from pregnancy related complications that will result in physical or mental disabilities [2–4]. The majority of these deaths and disabilities could be prevented with access to skilled birth attendants (SBA) and quality emergency obstetric care (EmOC) [5].

As most complications (often unpredictable) leading to maternal mortality and morbidity occur during the intrapartum period, delivery in health facilities has been advocated as a way to reduce maternal mortality as these complications can be appropriately managed by SBA and EmOC [6]. One-fifth of all global maternal deaths occur in India. The Indian Government adopted this approach of promoting in-facility birth to reduce maternal mortality through the implementation of a national cash transfer program, Janani Suraksha Yojana (JSY or ‘Safe Motherhood Program’), to incentivize poor women to give birth in a public health facility by providing them with a cash incentive ($23) upon discharge [7, 8].

In 2005–2006, when the program began, only 39 % of Indian women delivered in a health facility despite a number of investments by the government in the preceding decade to strengthen facility capacity [9–11]. The JSY in India is one of the first large scale conditional cash transfer (CCT) programs for maternal health that was implemented nationally by a government. Since inception to 2015, more than 105 million women have delivered under the JSY program [12, 13]. Eight years into its implementation, facility delivery in the country overall has risen to 74 % [14].

Despite the success of the JSY in raising institutional delivery proportions, the large number of beneficiaries, and the cost of the program, there have been few qualitative studies [15, 16] exploring why women participated (or did not participate) in the program. The program, though centered around the cash incentive, is supported by other initiatives such as community mobilization of women through the female village volunteers and the facilitation of transport to a facility [8, 17, 18]. While a common implicit (but unexplored) assumption has been that the cash incentive has contributed to the high program uptake, the role of the incentive or the other supporting elements of the program or the wider role of changing social norms that could influence program participation have not been studied.

We aimed to understand and interpret both JSY participants and non-participants’ experiences, perceptions, and motivations regarding place of delivery. In particular, we explored the role of the cash incentive and other elements of the JSY program, including community mobilization, to help elucidate reasons for delivering (or not) in public sector facilities.

## Methods

### Study context

The study was performed in Madhya Pradesh, a landlocked centrally located state in India. It is India’s largest state with a population of 72 million, 31 % of its population lives below the poverty line and 72 % live in rural areas [19, 20]. The state is divided into 51 administrative districts. Madhya Pradesh has seen a steep increase in institutional delivery since the implementation of JSY from 39 to 83 % [21]. However, JSY participation rates are not uniform across districts; between 2009–2011, the JSY participation rates varied from 49 to 88 % [21].

Two districts were purposively selected for the study. The first, located on the western side of the state, has a larger urban population, relatively better socio economic indicators and a flat geographical terrain. It has had a relatively high JSY participation rate (72 %) [21]. The second district on the other hand, lies on the eastern flank of Madhya Pradesh, is largely rural, has poor socioeconomic indicators, is densely forested and has seen a lower JSY participation uptake (59 %). Both districts were part of a larger project studying the JSY program [22]. Key characteristics of Madhya Pradesh and the study districts are presented in Table 1.

**Table 1:** Background characteristics of the two study districts and Madhya Pradesh

	District #1	District #2	Madhya Pradesh
Total Population (million) [19]	2	1.1	72
Rural Population (%) [19]	60	79	72
Female literacy (%) [19]	64	65	68
Human Development Index (HDI) [59]	0.626	0.564	0.375
Crude Birth Rate [21]	24	24	25
Institutional delivery (%) [21]	85	60	83
MMR [21]	176	361	227

### The Janani Suraksha Yojana program in Madhya Pradesh

In Madhya Pradesh, the public sector is the dominant provider of obstetric services; only 5 % of all births occurred in the private sector in 2013. The private sector is generally confined to cities (Sabde Y, Diwan V, Randive B, Chaturvedi S, Sidney K, Salazar M, et al. The availability of Emergency Obstetric Care under the JSY cash transfer program in Madhya Pradesh, unpublished). All care in private facilities is paid for out of pocket by the user/family. Women who deliver in a private facility therefore have high out-of-pocket expenditures compared to women who deliver in a public health facility where care is officially free to the user. The JSY in this state runs through the public sector institutions. The cash transfer program is operational through-out India and depending on the state, women have to meet different eligibility criteria to participate. In Madhya Pradesh, all women who deliver in public sector institutions are eligible for receipt of the cash transfer, regardless of parity or poverty status. The women in our sample did not differentiate between participating in the JSY program and delivering in a public health facility. For the purpose of this study we equate delivering in a public facility to participating in the JSY program.

The Accredited Social Health Activist (ASHA), as a trained female volunteer resident in each village, was envisaged and implemented under India’s National Rural Health Mission to support maternal and child health services [8, 23]. Under the JSY program, her key role is to accompany women to a public health facility for delivery [8]. Although each ASHA is considered a volunteer, she receives an outcome-based incentive for each woman she motivates for facility birth and accompanies to a public health facility [23]. There are currently 55,541 active ASHAs in Madhya Pradesh [18].

### Study participants

In-depth interviews were conducted in March 2013 with women who delivered within the last 12 months. A purposive sample of 28 mothers was selected, 14 from each district. Four declined to participate in the study citing lack of time as the reason. We included women to capture differences in age, parity and village distance from a health facility, as well as JSY participation. A list of JSY participants (and their home addresses) from a previous facility based study [17] was compiled. As part of the larger project survey, we had identified a cross section of program participants in each of the study districts. Using geographic information systems, we mapped the villages of residence of all of the identified women. A five kilometer radius was drawn around each village. Villages were then selected to include maximum geographical variation i.e. close/far from a road or a health facility, forested areas, remote and geographically difficult to access. We selected 11 different villages located across the two districts so that both remote and non-remote villages were selected.

The JSY participants were approached by a member of the research team. If the identified woman declined to participate in the study, a suitable replacement from the same village was identified and recruited. Non-participants included women who delivered at home or in private (non JSY) facilities. While the first author was interviewing the JSY participant, a research assistant had discussions with village inhabitants to identify women who delivered at home from the same village. Identifying these women was difficult in the first district due to the high JSY participation. Women who delivered in a private facility were identified using the same method as the JSY participant. Delivering in a private facility is uncommon in these two districts.

#### Characteristics of the study sample

The sample included 11 JSY participants and 13 JSY non-participants; three of whom delivered in a private facility and 10 who delivered at home (Table 2). Two-thirds of the women in the study sample were between the ages of 19 and 24 and half had little formal education (no or only primary level education). The majority of women had a low socio-economic background and came from vulnerable subpopulation groups (i.e. scheduled castes, tribes and other ‘backward’ castes^1^). Most women were multi-parous ranging from one to six previous deliveries, one quarter were primi-parous.

**Table 2:** Characteristics of participants by place of delivery in the two study districts

n = 24	Total	Public (JSY)	Private	Home
Age				
19-24	16	6	2	8
25-36	8	5	1	2
Education				
No/Primary Education	12	6	1	5
Secondary & Higher	12	5	2	5
Caste				
Scheduled Caste	5	1	0	4
Other Backward Caste	12	7	2	3
Scheduled Tribe	4	1	0	3
General	3	2	1	0
Parity				
Primi-parous	6	3	1	2
Multi-parous	18	8	2	8
Distance to EmOC Facility				
Close to facility (<5 km)	6	4	0	2
Far from facility (>5 km)	18	10	3	5

*EmOC* Emergency obstetric care

### Study instruments and data collection

Two semi-structured topic guides based on place of delivery were developed to conduct the in-depth interviews. At the beginning of the interview women were encouraged to share their pregnancy and delivery experience from the birth planning to after the delivery. The researcher probed when appropriate, specifically around their rationale for participating or not in the JSY program, facilitators for participation, barriers (where relevant) to desired participation or reasons for preferential non-participation of the program. Women were encouraged to be forthcoming with their experience and perception of the JSY program including the process in obtaining the incentive, its specific role in motivating women to deliver in health facilities and how it was used. The topic guides were aided by a previous study that quantified reasons for JSY participation and non-participation [24]. Both guides were translated into Hindi, back-translated and then subsequently piloted and refined.

Women were interviewed by the first author in Hindi at their place of residence with the assistance of a local interpreter from the study district. The in-depth interviews were conducted until saturation was reached [25]. Each interview lasted between 25 and 90 min and all interviews were recorded with consent. All interviews were transcribed in Hindi verbatim and then translated into English.

### Analysis

Qualitative framework analysis, a matrix based approach to structure and synthesize data, was used to analyze the data [26]. First a framework was developed to index recurring concepts and ideas within the data. After discussion, the research team agreed on a set of codes forming the initial analytical framework. Each code was given a brief explanatory description of its meaning to provide consistency during the coding process. The final framework consisted of 66 codes, clustered into 12 concepts (see web Appendix 1). The data was indexed in Microsoft Word 2010 with the codes, illustrating which concept applied where in the data. Three transcripts were dually coded by two different research team members to increase reliability. Participants’ responses (text) were charted within the thematic matrix using Microsoft Excel 2010. We viewed the data vertically through the thematic framework matrix and created category labels to highlight similar views, behaviors, and experiences. The category labels were then used to create the final themes and sub-themes.

### Ethical considerations and approvals

The study objective was described to each potential participant. Written or verbal informed consent was received before the interview began. Anonymity and confidentiality was ensured to all women. Ethical approval for the study was granted by the Ethics Committee of R.D. Gardi Medical College (Ujjain, India), Liverpool School of Tropical Medicine (Liverpool, United Kingdom) and Karolinska Institutet (Stockholm, Sweden).

## Results

Multiple factors influenced the women’s participation in the JSY program. As illustrated in Table 3, the first two main themes explore why women wanted to or did participate in the program while the last two main themes helped explain why women did not deliver under the program.

**Table 3:** Main themes, sub-themes and codes for why women participate or not in the JSY program

	Main Theme #1		
	Institutional delivery is now the social norm		
*Sub-theme*	*1.1 Social norm to deliver in* *a health facility*	*1.2 Individual women’s perception* *of the importance for ‘safe’ and* *‘easy’ delivery*	*1.3 Social pressure from the ASHA* *to deliver in a health facility*
*Codes*	Desired place of delivery · Justification for place of delivery · Affirmation of normalcy · Reflections on future delivery plans · Comparison between home delivery and institutional delivery · Advantages/disadvantages of home delivery and institutional delivery · ASHA involvement · *Dai* involvement · Anganwadi helper involvement · Sweeper involvement · How decision where to deliver is made · Who influenced · Role of Family members on where to deliver · Family relationships · Affirmation of normalcy · Reflections on future delivery plans · Perceptions of quality of care		
	Main Theme #2		
	Role of cash incentive: Diversity among views		
*Sub-theme*	*2.4 Incentive motivates for* *institutional delivery*	*2.5 Money is important but health* *is more important*	*2.6 Institutional delivery regardless* *of the cash benefit*
	*2.4.1 Difficulties to retain entire benefit and* *unintentional costs associated with* *participating in JSY*		
*Codes*	Influence of JSY on place of delivery · Reflections on future delivery plans · Justification for place of delivery · Awareness/View of JSY program · Expenses related to hospital delivery · Rationale for giving payments · Perception of delivery costs · Influence of JSY on place of delivery · Perception how the incentive should be spent · Adequacy of Incentive · Process/procedure to obtain incentive Actual use of incentive payment · ASHA involvement · Method of payment for delivery		
	Main Theme #3		
	Unintentional participation due to barriers to institutional delivery		
*Sub-theme*	*3.7 Circumstantial events and difficulties with transportation cause unintentional non-participation.*		
*Codes*	Pre-labor/labor experience · Transport experience · Role of transport in determining place of delivery · How decision is made · Role of Family members · Family relationships · ASHA involvement · *Dai* involvement · Role of Family members on where to deliver		
	Main Theme #4		
	Public hospital is acceptable for ‘normal’ delivery but not complicated		
*Sub-theme*	*4.8 Distrust in public delivery services*		
*Codes*	Trust/distrust in public sector to provide care · Trust/distrust in private sector to provide care · Comparison of sectors		

ASHA: Accredited social health activist, *JSY* Janani Suraksha Yojana

### Institutional delivery is now the social norm (Main theme #1)

#### Social norm to deliver in a health facility

The majority of interviewees’ accounts in the study, both JSY participants and non-participants, demonstrated a strong social norm towards facility-based deliveries and a shift away from a previous norm of home-based deliveries. Only one woman in our study sample preferred to deliver at home, but she acknowledged it was uncommon now and that most women delivered in a health facility. This norm is illustrated by these women’s comments:

*“I had decided to go to the hospital from the beginning [of my 4th pregnancy]. For the first three babies, I delivered at home. [For this one] I didn’t want to deliver at home. Nobody delivers at home now. All women go to the hospital…” – District 1 JSY Participant, Age 30*

*“Everyone in our village goes to the [public] hospital, that’s why I also went there.” – District 1 JSY Participant, Age 20*

Women particularly stressed a shift towards a commonly held perception of the risks of home delivery if complications occur:

*“Women have started changing their decision to deliver at home. Earlier women never thought like this. … People have started thinking that we should go to the hospital for better facilities and free delivery. There is risk in delivering at home.*

*You can’t get good treatment at home. If some women require blood during delivery, it is not available in village.... So, now women are aware and they want to go to hospital for delivery.” – District 2 JSY Participant, Age 23*

This perception was not only expressed among women who had an institutional delivery. The majority of women in our study who delivered at home also expressed the influence of a social norm to deliver in a health facility, even if they did not fully comprehend the clinical procedures that would take place in the facility.

The decision making process differed among the study participants; some described a process that involved several household members, while some of the older multi-parous women reported the ability to make their own decisions on where to deliver. Regardless of the individual decision making processes, all women agreed that the main decision-makers in their family supported institutional delivery in the context of strong social norms.

#### Individual women’s perception of the importance for ‘safe’ and ‘easy’ delivery

Shaped by the surrounding social norms, the vast majority of women voiced the necessity of delivering in a health facility to ensure ‘proper’ care was received during and after childbirth for both themselves and the baby. They also expressed personal desires for ‘a fast delivery’, ‘delivery to become easy’ and to ‘avoid problems’ as reasons for choosing a facility birth. However, in most circumstances, they could not articulate what constituted quality care further than ‘receiving all the amenities’ or ‘proper care’. Common elements of ‘proper’ care included ‘IV fluid’, which was linked with ‘giving strength’ and/or ‘a fast delivery’, and avoiding risk and/or complications.

One young mother from district 1 who delivered at home due to the unavailability of transport said, *“It is risky to deliver at home. Mother or baby can lose their life at home.”* Some women who delivered at home felt their health suffered as a direct result of the home delivery, affecting their perception on where they should deliver.

A few women expressed fear in relation to delivering in a hospital, especially primiparous women. They heard from other women that some are forced to undergo unnecessary (and unwanted) procedures and surgeries like cesarean section, episiotomy or sterilization. Many women also discussed feeling embarrassed and self-conscious about physical examinations. However, most felt this discomfort was worth enduring to ensure a ‘safe’ delivery.

#### Social pressure from the ASHA to participate in JSY

JSY participants often described the role of the accredited social health activist (ASHA) as instrumental in ensuring they delivered in a facility that participated in the JSY program. Whether the assistance was given during the decision making process or while arranging the practical logistics close to birth, the ASHA’s input constituted clear social pressure for facility use, contributing to the social norm in that direction. Interviewees generally viewed the ASHA as a resource to practically navigate the actual health care system. More specifically, the women found the ASHA ‘helpful’ in arranging their transport, accompanying them to the facility, facilitating their admission into the delivery ward and interacting with the facility staff on their behalf.

While many spoke of her influence in a positive or neutral manner, some women described ‘pressure’ from the ASHA to deliver in a facility. This perception was particularly strong among the group of women who unintentionally delivered at home. They frequently expressed a ‘fear of being scolded’ by the ASHA for not following her advice and instead delivering at home. This non-JSY participant was irritated by the ASHA’s reprimanding for having an unintentional home birth.

*“I became angry because she [ASHA] was scolding me…[ASHA said] You should have informed me and now hospital officials will say that I did not bring you in time. Delivery cannot happen so fast…”– District 1 Home Delivery, Age 21*

In summary, this study found that institutional delivery was an established social norm among the women and family elders. Individual women’s expressions of normative attitudes and beliefs regarding delivery commonly focused on avoiding risk and improving the ease of delivery. The ASHAs have also facilitated this social norm because of their insistence on facility delivery and the influence they have among the women.

### Role of the cash incentive: ambivalence towards the perception and influence of the incentive (Main theme #2)

There were three different perceptions towards the role of the JSY cash incentive in influencing women to deliver in facilities; (i) it was their main motivation for delivering in a facility, (ii) they acknowledged the assistance it gave in covering informal costs, but did not feel it was the main reason to deliver in a facility or (iii) they would deliver in a facility regardless of the benefit.

Around half of the women in this study reported that the JSY cash incentive motivated them to have an institutional delivery. At the beginning of the interview women were usually hesitant to speak regarding the role of the financial incentive in their decision. By the end of the interview, however, they clearly stated their preference for an institutional delivery was based on receiving the financial incentive. To further corroborate this perception, we asked them to rank what they viewed as advantages to delivering in a facility. Often their first or second response was receiving money from the government. As illustrated below, a woman who delivered at home displayed regret at not participating in the program and ultimately not availing the cash benefit:

*“I always wanted to deliver in the hospital as I will get money. But I was unable to go to hospital for delivery. I was in field harvesting crops during this delivery and suddenly the baby came out. What could I do? … I thought that it would have been better if I delivered in hospital because in hospital we get the money… At home you get nothing.” – District 2 Non-JSY Participant (Home delivery), Age 30*

In addition to not availing the cash benefit, some non-participants felt a home delivery forced them to accrue more debt than if they had delivered in a facility. They described borrowing money ‘to pay the *dai* (traditional birth attendant*)* and others’, whereas they felt a delivery in a public health facility would have been free. They viewed the program as a mechanism to enable facility deliveries by providing money to cover the out-of-pocket expenses and avoid costs incurred with a home birth.

*“[I wanted to deliver my baby] in the hospital but previously thought to deliver at home because at the hospital the nurse … will take money (Rs.200, $3.33) so it becomes a problem. Now in the hospital, we get money for delivery … we will get money to help pay for all the expense but not at home.” District 2 Non-JSY Participant (Home delivery), Age 23*

While a couple of JSY participants reported receiving the cash benefit upon discharge, most described difficulties in *obtaining* the cash incentive, including procedural hurdles of opening a bank account to deposit the check and being instructed to come back to the health facility at a later time to retrieve it. They also recounted trouble *retaining* the entire benefit due to having to pay rewards to hospital staff and the ASHA, pay for medicines, fees to open the bank account and transportation related expenses. All JSY participants reported at least some out-of-pocket (OOP) expenditure. The incentive was generally large enough to cover the OOP (largely informal payments to staff), however most women reported having a small sum remaining after paying for the expenses mentioned above. The women knew that the hospital staff was not supposed to ask for money but they felt powerless to refuse to pay for fear of not receiving ‘proper’ care.

Some women conveyed conflicting emotions when talking about the costs associated with a facility-based delivery and receiving the benefit. They questioned the appropriateness of having to pay for some of the delivery services, but felt the program provided cash to pay for these elements thus avoiding additional OOP expenditures and accruing debt. In contrast, some women reported the expenses were paid by her family, while the cash incentive was collected by her husband’s family thus the expenses and the benefit did not cancel each other out.

Regardless of the impediments they described to get the benefit and their feelings towards the OOP payments, when asked how this would affect their decision to participate in the program in the future, all but one JSY participant replied it would not have an impact.

Some women acknowledged the benefits of receiving the cash incentive, but stated it was not the principal motivation behind their decision to deliver in a public health facility. Further, when asked what would happen if the government stopped the JSY program, they expressed the importance of health over accumulating debt and said they would still prefer to deliver in a health facility.

There were also women, mostly JSY participants but some non-participants, who explicitly said the role of the subsidy did not factor into their decision on where they should deliver. A JSY participant expressed this sentiment;

*“We will go to the hospital even if we don’t get the money because we go to the hospital for better health and good treatment. Not for the money. If we die without good treatment, what we will do with the money?” – District 1, Age 28*

In summary, for around half of the women interviewed, the cash incentive was an important motivation to deliver in a facility. However they reported procedural difficulties with obtaining the money. For some women it helped defray expenditures incurred in connection with the childbirth, whilst for others it did not. Yet a few women reported that the cash incentive was not important in their decision to deliver at a facility.

### Why do women not participate in the program?

Main themes three and four (Table 3) emerged to help explain why women did not participate in the program. Main theme three highlights that most home deliveries were not intentional. It also captures the women’s experiences with barriers to institutional delivery, fast labor and delivery, arranging transport, or no one to accompany them. The fourth explores the perception that government hospitals are not equipped to handle complicated deliveries. Regardless of the money, when there is a perception of a problem that could jeopardize their health, they will go to a private facility to deliver, if possible.

### Unintentional non-participation due to barriers to institutional delivery (Main theme #3)

All but one of the women who delivered at home expressed intent to deliver in a health facility that participated in the JSY program. These women were not able to participate due to circumstances outside of their control. More than half of the women reported that the baby came ‘too fast’ and delivered before they tried to arrange transport. A few women reported they did not have anyone to accompany them to the health facility during a night delivery, for example:

*“I never decided to deliver at home. But it is difficult to reach hospital at night as my husband was not at home … I decided that if there is pain during the day I would go to hospital but if pain is there at night I would do it at home. During the day I can go alone or call ASHA worker. But at night who will run and call ASHA or the vehicle? – District 2 Non-JSY Participant (Home Delivery), Age 22*

Another woman recounted how she contacted the ASHA prior to delivering, but due to a combination of factors (labor starting at night, poor weather and road conditions); it was not possible for her to access a health care facility for delivery. A couple of women experienced the more common access barriers to institutional delivery like trying to arrange transportation, for example:

*“I planned to deliver in the hospital…When the pain started it was raining heavily. When I told my parents about the pain, they called the [free emergency transport] vehicle but they were not able to get through because of the heavy rains…We were planning to go to the hospital but the baby came out very quickly.” – District 2 Non-JSY Participant (Home Delivery), Age 22*

### Public hospital is acceptable for ‘normal’ delivery but not complicated (Main theme #4)

Around half of the women were content to deliver in the public sector if they felt healthy and thought the delivery would be ‘normal’. Otherwise they would prefer to seek care from a facility offering what they perceived to be a higher quality of care in the private sector. This ‘common sense’ norm was expressed among JSY participants and both groups of non-participants (i.e. women who delivered at home or in a private institution). When asked where this JSY participant would deliver if she became pregnant again, she succinctly said,

*“We will go to [public] hospital but if I feel very weak, I will go to the [private] facility.” – District 1 JSY Participant, Age 20*

This perception was also reinforced by other community members. One woman, whose first pregnancy was complicated, explained how the *dai* planned to take her to the government hospital, but if there was a complication the *dai* would encourage her to immediately seek care at a private facility.

## Discussion

Over the last decade, several health interventions have included a monetary component to increase utilization of services while simultaneously addressing financial access barriers, such as the cash incentive in the JSY program. Results from quantitative evaluations show a significant relationship between increased institutional delivery and the JSY program indicating the cash incentive has been successful in changing women’s behavior to seek institutional delivery [27–30]. However, there are multiple pathways influencing participation in a program like JSY as the program (a) has a number of supporting elements besides the monetary incentive i.e. ASHA and transportation support and (b) the program does not occur in a vacuum but in a context with dynamic social norms around childbirth.

To summarize our findings, we found that there has been a shift in the norm on where women should deliver. Under this social norm, women expressed their individual perception of the importance for a ‘safe’ and ‘easy’ delivery. Women also discussed pro-social pressure from the ASHA and *dai* to have an institutional delivery.

The women expressed diverse views on the role the cash incentive played in their motivation to deliver in a government operated facility. Around half of the women clearly described the incentive as an enabler and felt it was the sole reason to deliver in a health facility. Just as many women felt it did not play a role in their decision to deliver under the JSY program. A few appreciated the small financial support the incentive provided, however did not feel it was the main reason to seek an institutional delivery.

Women also described challenges in the process to participate in the JSY program, including difficulties in obtaining the cash benefit and unanticipated costs associated with participating in the program. However in our study these issues, although concerning, did not appear to affect their rationale or future intent for delivering in a health facility. Unintentional home deliveries and the perception that public facilities were not competent to manage a complicated delivery were the main reasons women did/would not participate in the program.

### Social norm to delivery in a health facility and the ASHA’s role

Social norms are unwritten rules of conduct that dictate and influence interactions among individuals within a specific reference group [31, 32]. It has been previously shown that community beliefs and norms in reference to health behaviors strongly influence the decisions made by individuals to seek care [33]. In our study almost every woman we interviewed mentioned a shift in social norms that has occurred away from delivering at home to delivering in a health facility. Further, given the choice now, women in Madhya Pradesh do not usually opt to deliver at home. Institutional delivery has become normalized to such an extent that women who have home deliveries are perceived as deviating from the norm.

While JSY has likely contributed to making institutional deliveries the norm, it has also probably simultaneously harnessed this new emerging social norm to encourage and increase participation. Several women stated their rationale for going to the public health facility was based on what others in her community were similarly doing. Since individuals habitually emulate what others do, the diffusion of this social norm throughout the community has been a pivotal component to the high participation rate of JSY.

The ASHA has previously been characterized as an ‘agent of change’ [34]. From the perspective of the women in our study; the ASHA is an agent of change by promoting and perpetuating the new social norm that we described above on behalf of the community and the government. Due to her position of relative authority as a quasi-government agent, her advice is often followed. Many of the JSY participants in the study described her role in a neutral or positive manner and felt they had an advocate who could navigate the often stressful situation in the health facility.

However, some of the women in our study expressed concern or fear of retribution from the ASHA for an unintentional home delivery. One study found that external pressure by the ASHA contributed to the increase in JSY participation [35]. Although this is probably another important component to the high participation rate, some have argued the JSY program exerts strong/potentially coercive pressure on women’s behavior rather than sufficiently recognizing and supporting women’s agency and rights [36, 37]. Studies have reported that ASHAs cite their financial incentive, Rs. 600 ($10) for every woman they accompany to a public health facility for delivery [8, 38], as the main motivating factor for their activity. Therefore the social pressure they exert on the women is probably motivated by the outcome-based incentives they receive [37, 39].

To understand how the social norm shifted and how interventions can be designed more effectively to harness such changes, the individual must be seen as embedded in a larger social system, i.e. her community. In this regard, the JSY program is an example of how maternal health programs can be designed to not only influence individuals’ behavior but also aim to catalyze change at the community level. It is crucial to understand what perpetuates such norms and how norms can be transformed by different kinds of interventions. While our research identified a shift, we do not know *how* it was formed. Responses indicate that the JSY program itself had some effect in establishing the norm, through the ASHAs. Further research is needed to understand other factors that have shaped these norms.

### Individual perception of safe and easy delivery

The women in our study express their rationale for participating in the JSY program by describing their personal belief in its importance for a ‘safe’ and ‘easy’ delivery. More et al. also found this as a reason to have an institutional delivery among women living in an Indian slum [40]. This likely reflects individual perceptions and expressions of the normative rationale for institutional delivery. Especially since the women in our study demonstrated limited understanding of what constitutes actual quality of care, so their decision to seek an institutional delivery may not be based on a rational decision through informed choice but more influenced by a strong social norm.

While the English translation of JSY means ‘safe delivery’, it is important to note that institutional delivery does not necessarily equate a safe delivery, especially in this setting. The underlying assumption of JSY is if a woman has an institutional delivery it will be attended by skilled health personnel and she will receive the appropriate care thus reducing the likelihood of a maternal death. There have been several reports questioning the quality of care administered in the government operated facilities [17, 41–44], where the majority of women in this study and in Madhya Pradesh avail birth services. When access to affordable *quality* health services is lacking, addressing only utilization of services is unlikely to have any impact on maternal outcomes. Others have commented this may be a factor in explaining why the maternal mortality ratio has not declined as expected in line with the higher institutional delivery rates [17, 28, 41, 43]. In this scenario, addressing the supply side issue needs to be a priority and happen simultaneously with increasing demand for services.

### The role of the cash transfer in determining place of delivery

In the 90′s CCTs were introduced in Latin America to modify behavior with regards to improving access to preventative medicine, school attendance or nutrition. Similar CCT programs have since been implemented in sub-Saharan Africa and South Asia and expanded to improving uptake of maternal health services. There is extensive research demonstrating CCTs increase utilization to the desired service [45], however there are very few studies focusing on *why* the programs are successful and the role the monetary incentive plays in this modified behavior.

The JSY intervention design is based on the classic economic assumption that people’s behavior is rational. Specifically, that it can be influenced by financial incentives when complemented by information and education. While the results from quantitative evaluations of JSY indicate a degree of justification for this assumption, to our knowledge there have been no studies focusing on *why* JSY is effective. Adato et al. argue that the impact of a cash incentive is more limited than originally assumed by CCT programs, but it does have the potential to instigate behavior change. They also contend that sociocultural drivers (such as gender and social norms, roles and relations) strongly affect the way people respond to a program like JSY and are equally important to consider when trying to understand why people participate in a specific program [46].

A randomized control study on incentives to assist in smoking cessation found that although the cash incentive intervention group was significantly more likely to quit smoking, participants denied the incentive played a role. Volpp et al. described the tendency people have to attribute behavioral change to their own motivations versus a monetary incentive [47]. We also found in our study that JSY beneficiaries were less likely to attribute the JSY program to their seeking an institutional delivery, while the women who delivered at home explicitly stated it was a main motivator. The complexity behind modifying health seeking behavior, sociocultural drivers, and the intrinsic desire to attribute a perceived ‘healthy’ change to one’s self could possibly explain the diversity in views towards the cash incentive in our study. The strength of the new social norm as a main motivator may be another. These issues have rarely been explored in studies of CCTs, especially in low- and middle-income settings.

Some of the JSY participants in our study described several obstacles to obtain the cash incentive and often reported waiting two to three months to receive the incentive. One of the most attractive features of a cash incentive program to alter behavior is the immediate benefit of receiving cash [48]. This becomes even more important if the cash is needed/expected to pay for hidden costs at the hospital, transport and other costs that would benefit the mother such as nutritious food in the post-natal period. While many women have probably come to expect these types of delays, potential risks remain to the future outcomes of JSY and other programs if trust in the distribution of incentives is undermined. The literature on other CCTs does not explicitly discuss the program utilization impact of these issues [45], however it could be important for the sustainability of program to address these bureaucratic procedural issues, and ensure women receive the money with relative ease and in a timely manner.

Delivering under the JSY program is supposed to be cashless [8]; however all the beneficiaries in our study reported OOP expenditures. While they did not feel the additional OOP expenses outweighed the benefit of receiving the cash incentive and of having a trained birth attendant, they were not pleased to pay it. From our interviews it seems there is equilibrium between the incentive and the amount of money paid to the health facility, so families are neither earning nor losing a significant amount of money by participating in the program. While studies have found that OOP in the public sector have decreased over time [49], attention is still needed to address OOP and decrease the occurrence of informal payments [50]. Particularly in the context of cash transfer program, so that the cash incentive stays with the woman and is not used to defray informal expenses at the facility.

### Non-participation is largely unintentional

Our results showed despite the best efforts of community health workers and the women with their families, some unintentional home deliveries occurred. The most common reasons for a home delivery given in our study were generally not related to health system deficiencies or structural access barriers but were often circumstantial. We also found that women rarely intended to deliver at home. There were a few cases that reported experiencing difficulties arranging transportation; however most alluded to the speed of the delivery as the main barrier to participating in the program. A previous study conducted among women who delivered in 2009 from the same area reported non-availability of transportation as the main underlying reason for home deliveries [24]. In light of the implementation of an emergency transport intervention [17], it would appear the reasons for home deliveries in this area has shifted away from health system based access barriers like transport and towards more individual circumstantial situations.

While previous studies have shown that financial access barriers are often responsible for not seeking an institutional delivery [16, 51], none of the women in our study reported economic related issues as the reason for their home delivery. This does not mean the financial access barrier has been completely eliminated in this context; but it has mostly likely been significantly diminished. Unpublished data from a community based study [22] corroborate our finding regarding the speed of delivery as the most common reason for home deliveries. Precipitate labor has been described in 2% of pregnancies [52]; the program can exert little influence on ensuring these births also occur in a facility.

Perceived quality of care is an important factor influencing where women want to deliver. Studies have found women prefer to deliver in a private facility when confronted with a poorly functioning public health sector [53–55]. While the women in our study did not explicitly say this, they did express doubt at the public health facility’s ability to manage complicated deliveries. The women’s own perception of quality and improving the quality of care may be a key element to increase participation in the program. The women in our study were not able to clearly articulate their understandings of good or better quality of care, but this perception is widespread and probably reflects dissatisfaction with the care received in public facilities [56].

### Methodological considerations

Interviewees were purposively sampled for maximum variation to represent a range of characteristics of women giving birth in rural Madhya Pradesh, including areas where participation in the JSY program was both relatively low and high. Credibility is increased as they should reflect the range and types of perspectives of this population [57]. However, they are not intended to statistically represent the distribution of views or experiences among individuals in the districts, state or country.

To increase trustworthiness we used different methods that included researcher triangulation during the coding process, developing the themes, and peer debriefing. The authors involved in this study have diverse backgrounds from medicine, social science, public health and economics. By incorporating researchers with different disciplines, we have been able to provide varying perspectives to improve the understanding and interpretation of the data.

We were aware of the young women’s relatively low status position in the household and how the presence of others could deter them from expressing their true thoughts and opinions. Whenever possible, we asked to speak to interviewees on their own, participants were encouraged to speak openly and freely and it was emphasized several times that there was no right or wrong answers.

‘Mutedness’ is the concept that the least powerful tend to internalize norms supported by the more powerful [58]. Young women’s subjectivity may be such that they find it difficult to perceive, let alone express their own needs and opinions. The women in our study could have been projecting the perceived social norm and not their own personal beliefs. This is relevant in this setting given the women’s position in their family and community. It is an important concept that should be taken into consideration when conducting this kind of research.

We did not explore the extended family’s perception of the cash program. Considering the strong gender norms/relationships in India and unique power dynamics between the woman and her marital family, it would be important to explore the family’s perception of the incentive to fully understand its influence on the women’s choice.

We acknowledge the barriers and reasons for the unintentional home delivery in our study could differ from the women who intentionally delivered at home. Several attempts were made to recruit these women, however despite including fairly remote villages we were unable to find more than one woman within the time for the study.

## Conclusion

Our research found the decision of most women in this study to participate in the JSY program reflects a change in social norms towards delivering in an institution. These women expressed an individual perception of the importance for ‘safe’ and ‘easy’ delivery which was most likely an expression of the new social norm. Social pressure from the ASHA to deliver in a health facility also contributed to the social norm. Many women reported their behavior was influenced by receiving the incentive, but just as many said it did not play a role in their decision to deliver in a facility. There were limits to the influence of the cash and behaviors may be as much influenced by social norms and social pressures for many. Non-participation was often unintentional due to personal circumstances, geographical access issues or driven by a perception of poor quality of care in public sector facilities. In summary, while the cash incentive was important for some women in facilitating an institutional birth, the shift in social norm (possibly in part facilitated by the program) and therefore their own perceptions has played a major role in them giving birth in facilities.

## Appendix 1

**Table 4:** Concepts and codes used during the analysis

Concepts/Code	Description
*1. Birth Preparation*	
Source of information for delivery	Who or how the women come to know about where to deliver or gain information about the programs
Birth prep/reasons	No preparation, documents needed, clothes for the child and mother, arranging the money for the delivery
Geographic birth prep	Where did they go to get ready to deliver - natal/affinal home
*2. Decision-making on place of delivery*	
How decision is made	The process involved for making the decision on where she should delivery
Who influenced	Who was involved in influencing the mother on where she should deliver (family/health worker/ASHA)
*3. Place of delivery*	
Desired POD	[place of delivery] Where they think women should deliver, and where they wanted to deliver regardless of where they actually delivered
Justification for pod	[place of delivery] Why they delivered where they did ^*a*^ *Do not include if they stop JSY* ^*a*^
Affirmation of normalcy	Pertaining to the delivery. If everything was “normal” they would just deliver x
Home vs ID	[Institutional delivery]. Comparisons between home and facility deliveries.
Advantages of HD	Home deliveries – benefits related to delivering at home, in general for all women, specifically to her
Disadvantages of HD	Home deliveries – drawbacks related to delivering at home, in general for all women, specifically to her
Advantages of ID	Institutional deliveries – benefits related to delivering at a facility, in general for all women, specifically to her
Disadvantages of ID	Institutional deliveries – drawbacks related to delivering at a facility, in general for all women, specifically to her
*4. Labor/delivery Narrative*	
Pre-labor/labor experience	Experience of pre-labor/intrapartum period, recognition of labor pains, the labor “story”
Hospital staff interaction	Actual interaction with the staff at the hospital, who took care of them, what did they say or how did they make the women feel
Power relationship between staff and mother	
Reflections on future delivery plans	Based on the current experience, where would they like to deliver if they became pregnant again
Affirmation of normalcy (labor)	
Perceptions of QOC	[quality of care] (in general, what is good care/bad care)
*5. Social and personal context of childbirth*	
Home environment	The dynamic at home that would influence her decision on where to deliver
Beliefs around childbirth	
Son preference	
Family relationships	How they influence her pod
Social norms of home vs institutional delivery	
Role of Family members	In pregnancy/birth/PNC (e.g. ANC visits/transport/go for PNC)
*6. Emergency Transport*	
Transport experience	The women’s experience gaining access to the transport, how they travel to the facility, difficulties encountered gaining access
Role of transport in determining POD	[place of delivery] How did transport (gaining access to influence where they ultimately delivered)
Type of transport used	Descriptive–specifically the type of transport used to travel to the facility
Who arranged transport	The person responsible for arranging transportation to the facility
Reasons for type of transport	Justification for why they used one type of transport over another ie Reliability
Awareness of JE	Awareness of Janani Express for transport
Perception of JE	Perception of using the service for transport
*7. Role of community members*	
* Dai*	(role/trust in)
Anganwadi helper	
Sweeper	
ASHA	Perception of ASHA and what she should do in her role, Pressure from ASHA to have an ID delivery, involvement during ANC, intrapartum and PNC
*8. Ante Natal Care*	
ANC visits	Use/reason/Place for ANC visits. Descriptive–what did she do
Affirmation of normalcy (ANC)	
*9. Post Natal Care*	
Use of PNC	
Reasons of PNC	Include reasons not to have PNC
Affirmation of normalcy	
*10. Expenses*	
Expenses related to hospital delivery	Transport costs, money paid to health workers
Rationale for giving payments	
Expenses at home	
Perception of delivery costs	Home/hospital/with or without scheme
Method of payment for delivery	If loan, repayment schedule etc; implications, when the loan was taken
*11. Government Programs*	
Awareness/View of JSY program	How they came to know about the program, adequacy of incentive, perception of the program
Perception how the incentive should be spent	The ideal use, why the money is given to the women
Actual use of incentive payment	
Experience of JSY	Process to gain incentive/procedural hurdles etc
Awareness/perceptions of other government schemes	Corruption of government services/programs
Influence of JSY on POD	[place of delivery] (decision-making)
*12. Trust*	
Trust/distrust in the Public sector to provide care	
Trust/distrust in the Private sector to provide care	
Comparison of sectors	(e.g. perception of differences)

^a^Concepts are numbered 1-12, codes are beneath respective concepts

## Abbreviations

ASHA: accredited social health activist
CCT: conditional cash transfer
EmOC: emergency obstetric care
JSY: Janani Suraksha Yojana
OOP: out-of-pocket
SBA: skilled birth attendants

## Footnotes

1 ‘Backward’ caste, scheduled caste and tribe are groups of people historically subject to social disadvantage. They were awarded special status by the Constitution of India that entitles them to specific social benefits [60].
